# Nitrate and Nitrite Variability at the Seafloor of an Oxygen Minimum Zone Revealed by a Novel Microfluidic In-Situ Chemical Sensor

**DOI:** 10.1371/journal.pone.0132785

**Published:** 2015-07-10

**Authors:** Mustafa Yücel, Alexander D. Beaton, Marcus Dengler, Matthew C. Mowlem, Frank Sohl, Stefan Sommer

**Affiliations:** 1 GEOMAR Helmholtz Centre for Ocean Research, Kiel, Germany; 2 Middle East Technical University (METU), Institute of Marine Sciences, Erdemli, Mersin, Turkey; 3 National Oceanography Centre Southampton, Ocean Technology and Engineering Group, Southampton, United Kingdom; 4 DLR German Aerospace Center, Institute for Planetary Science, Berlin, Germany; University of California, Merced, UNITED STATES

## Abstract

Microfluidics, or lab-on-a-chip (LOC) is a promising technology that allows the development of miniaturized chemical sensors. In contrast to the surging interest in biomedical sciences, the utilization of LOC sensors in aquatic sciences is still in infancy but a wider use of such sensors could mitigate the undersampling problem of ocean biogeochemical processes. Here we describe the first underwater test of a novel LOC sensor to obtain in situ calibrated time-series (up to 40 h) of nitrate+nitrite (ΣNO_x_) and nitrite on the seafloor of the Mauritanian oxygen minimum zone, offshore Western Africa. Initial tests showed that the sensor successfully reproduced water column (160 m) nutrient profiles. Lander deployments at 50, 100 and 170 m depth indicated that the biogeochemical variability was high over the Mauritanian shelf: The 50 m site had the lowest ΣNO_x_ concentration, with 15.2 to 23.4 μM (median=18.3 μM); while at the 100 site ΣNO_x_ varied between 21.0 and 30.1 μM over 40 hours (median = 25.1μM). The 170 m site had the highest median ΣNO_x_ level (25.8 μM) with less variability (22.8 to 27.7 μM). At the 50 m site, nitrite concentration decreased fivefold from 1 to 0.2 μM in just 30 hours accompanied by decreasing oxygen and increasing nitrate concentrations. Taken together with the time series of oxygen, temperature, pressure and current velocities, we propose that the episodic intrusion of deeper waters via cross-shelf transport leads to intrusion of nitrate-rich, but oxygen-poor waters to shallower locations, with consequences for benthic nitrogen cycling. This first validation of an LOC sensor at elevated water depths revealed that when deployed for longer periods and as a part of a sensor network, LOC technology has the potential to contribute to the understanding of the benthic biogeochemical dynamics.

## Introduction

In situ, high frequency observations are crucial to uncover the temporal and spatial complexity in the aquatic environments [1]. Considerable progress has been made with the increasing use of in situ sensors on a variety of platforms; however these devices mostly address physical parameters such as temperature, light or pressure [2, 3]. On the other hand, with the notable exception of oxygen [4,5], pH [6] and nitrate at low (μM) resolution (see below), routine calibrated and accurate measurements of chemical parameters still depend on spatially and temporally limited sampling schemes and subsequent analyses onboard research vessels or in shore-based laboratories. Hence, the biogeochemical variability of oceanic habitats is so far largely unknown but is ultimately important to understand the productivity, elemental cycles and ocean response to a changing global climate.

As a limiting nutrient of biological productivity, nitrate (NO_3_^-^) and nitrite (NO_2_^-^) are among those parameters for which several sensor technologies already exist, such as a UV-spectrophotometry based system reported by Johnson et al. [7] (also see [8, 9]), and the colorimetric system by Le Bris et al. [10] (for reviews see [11–13]). Some nitrate sensors have become commercially available (such as Satlantic ISUS, Systea WIZ), and recent applications of these sensors uncovered the high temporal variability in NO_3_^-^ in the open ocean [8]. The temporal variability can be even higher in estuaries, where current can be fast and freshwater inputs are significant [14]. Despite these improvements, the capacity of many nutrient sensors to perform long term, stable measurements at elevated water depths may be limited due to shallow depth range, large size, large power consumption and/or high detection limits.

The bottom waters of oceanic oxygen minimum zones (OMZ) are one of those environments for which there is almost no information on the short-term temporal nutrient variations. Here, the availability of NO_3_^-^/NO_2_^-^ drives the anaerobic degradation of organic matter in the water column and the sediment surface [15]. Moreover, these electron acceptors are involved in the anaerobic oxidation of NH_4_^+^, which is released in high amounts from the seafloor under anoxic conditions [16, 17]. In organic-rich sediments NO_3_^-^/NO_2_^-^ can be further involved in the oxidation of sulfides [18]. Besides consumption and production, physical transport processes such as currents, submesoscale eddies or passage of internal waves can also drive the variability of these nutrients over continental shelves below OMZs [19].

To accurately constrain the variability of NO_3_^-^/NO_2_^-^ at the seafloor of an OMZ, we need pressure-insensitive, robust sensors with the capability of in situ calibration. The in situ calibration is particularly important for an application at a fixed point at the seafloor, where there is potential for variability over short time scales. In this regard, microfluidics, or ‘lab-on-a-chip’ (LOC) is a promising technology that meets several demands needed for autonomous measurements at elevated depth such as low weight, low energy demand, and low volume consumption of reagents and samples [20, 21] and ease of integration into underwater platforms. Having been mostly developed for medical and pharmaceutical research [22], LOC technology is now being transferred to aquatic environmental research as well [23–27]. Newly developed LOC devices can measure not only NO_3_^-^ and NO_2_^-^, [23, 26, 27] but also PO_4_^3-^ [28] and dissolved Fe and Mn [29]. Among these devices, the NO_3_^-^ / NO_2_^-^ LOC system is approaching maturity for time-series applications but, prior to the work reported here, its suitability for the in situ deployment at elevated depth has not been demonstrated.

Using a new generation LOC sensor that was capable of in situ calibration, we report the first in situ time series (up to 40 h) of NO_3_^-^+ NO_2_^-^ (ΣNO_x_) and NO_2_^-^ at the seafloor (max. 170 m depth) in an upwelling region offshore Western Africa. We combine these data with time series of oxygen, temperature and bottom currents to demonstrate that the LOC technology provides a previously unavailable window into the variability of nutrient concentrations in the bottom waters of a low-oxygen coastal ocean.

## Methods

### Study Area

The study site was near 18°N in the Mauritanian upwelling region which is part of the Canary eastern boundary upwelling system that roughly extends between 43°N to 10°N [30, 31]. Fieldwork permit was obtained from Mauritanian Ministry of Fisheries and Maritime Economy (*Ministère des Pêches et de l'Economie Maritime*, Permit No: 296, dated May 14, 2014). Coastal upwelling near 18°N off Mauritania exhibits a pronounced seasonality where winds favorable to upwelling prevail primarily from December to April. Due to the weak mean circulation in the eastern tropical Atlantic, an oxygen minimum zone is situated below the surface layers. While the core of the main OMZ is found at about 400 m depth, a secondary oxygen minimum is situated below the surface mixed layer and above 200 m depth [32]. The ventilation of the waters above the continental margin occurs primarily through the Mauritania Current [33] in the near-surface layers and the Poleward Undercurrent below [34]. Both currents transport relatively oxygen-rich South Atlantic Central Water, which is supplied by the eastward flowing North Equatorial Countercurrent and North Equatorial Undercurrent, northward into the upwelling region.

A physical-biogeochemical measurement program was performed during the R/V Meteor cruise M107 from May 28 to July 3, 2014 during the termination period of the upwelling season as a part of a collaborative research center Climate-Biogeochemistry Interactions in the Tropical Ocean (SFB754) and the Helmholtz-Alliance ROBEX (Robotic Exploration of Extreme Environments). The observational program included benthic lander deployments, conductivity-temperature-depth-oxygen (CTD/O_2_) profiling paired with water sampling and mooring deployments measuring currents and hydrography along a transect at 18°N.

### Lander deployments at the Bottom Boundary Layer

A GEOMAR benthic lander (thereafter as the Lander) was used as a platform to make autonomous in situ measurements [35]. The Lander was deployed on the sea floor at depths of 50, 100 and 170 m (Table 1). These depths were chosen as bottom water oxygen time series displayed high variability during a previous cruise [36] (R/V MS Merian leg 17/4) and benthic nitrogen turnover rates were high due to hypoxia [17]. At all locations, the LOC sensor (Ocean Technology and Engineering Group, National Oceanography Centre Southampton) was attached to the lander and at the 50 and 100 m sites this LOC system was complemented with a CTD probe (RBR, Ottawa, Canada) and an O_2_ optode (Aandrea, Bergen, Norway). For the deployment at the 50 m site an additional LOC sensor measuring NO_2_^-^ was added. Prior to the Lander deployments, the LOC sensor was tested in situ on the CTD rosette to compare the sensor results to concentration measurements from water samples analyzed on-board using an autoanalyzer (Quattro, Seal Analytical, UK).

**Table 1:** Details of underwater operations during R/VMeteor cruise M107 where autonomous measurements were performed.

Depth (m)	Operation	RV Meteor Station	Longitude (N)	Latitude (W)	Measured Parameters	Measurements Start (2014, UTC)	Measurements End (2014,UTC)
50	Lander 3	M107-687	18°17.0'	16°19.0'	NO_3_^-^+ NO_2_^-^, NO_2_^-^, O_2_, CTD	June 25, 15:00	June 26, 23:00
100	Lander 2	M107-633	18°14.7'	16°27.0'	NO_3_^-^+ NO_2_^-^, O_2_, CTD	June 21, 15:00	June 23, 07:00
170	Lander 1	M107-572	18°14.2'	16°31.0'	NO_3_^-^+NO_2_^-^	June 13, 16:00	June 14, 14:00
170	CTD #16	M107-559	18°14.0'	16°31.0'	NO_3_^-^+NO_2_^-^	June 12, 15:00	June 12, 17:10
50	POZ Lander	M107-505	18°16.0'	16°19.0'	Current velocity	June 8, 18:00	June 27, 07:00
50	BIGOII-4 Lander	M107-665	18°17.1'	16°19.0'	NO_2_^-^, syringe samples		

### Application of the Lab-on-Chip Nitrate/Nitrite Sensor

The LOC ΣNO_x_ sensor was previously described in detail by Beaton et al. [23, 26]. That paper as well as Ogilvie et al. [37] and Floquet et al. [38] described various aspects of the lab-on-a-chip approach for nutrients. In short, the chip used in the sensor was made up of PMMA and harbored precision-milled microchannels (< 300 μm), mixers and optical components such as LEDs (525 nm) and photodiodes. An integrated syringe pump, valves and electronics complemented the chip and are encased in mineral oil-filled housing (PVC, 12 cm diameter, 30 cm height) with an internally fitted pressure-compensating bladder. The system ran autonomously storing the data in a memory card and was powered by an external battery. The unit weighed 1.1 kg in water without and 1.5 kg with an internal battery.

The sensor uses attached standards and blank for regular in situ calibrations along with sample (0.45 μm filtered) measurements, using the colorimetric Griess assay for NO_2_^-^ detection [39]. The addition of an off-chip Cu-activated Cd column enabled NO_3_^-^+NO_2_^-^ (ΣNO_x_) detection through the reduction of NO_3_^-^ to NO_2_^-^ and subsequent on-chip analysis with a detection limit of 20 nM, an accuracy of 0.6% and a precision of 7 nm at low concentrations or 0.5% at high concentrations [23]. The deployment involved externally attached gas impermeable Flexboy bags (150 mL, Sartorius, UK) that contained two standard solutions (for either ΣNO_x_ or NO_2_^-^, as needed), Griess reagent, artificial seawater blank and imidazole buffer. The preparation of the standards and reagents followed previous protocols [40, 23, and 26]. Waste was collected in a 500 mL Flexboy bag. For example, for a deployment to measure ΣNO_x_, the sensor started to perform two sets of calibrations (artificial seawater blank and 11.3 and 33.9 μM standards for NO_3_^-^) followed by a repeating sequence involving the measurement of blank, sample, standard solution and sample again. Each of these steps included 6 flushing cycles to avoid any carryover. The final flush cycle was followed by a 100-second waiting stage that enabled color development and the photometric measurement at 525 nm. A set of flushes and the waiting stage altogether took about 7 minutes. The sensor recorded optical absorbance every 1 second and the average of the last 3 reading of the waiting stage was used for calculations. Raw data were processed in R 3.0.2 [41]. With this scheme, we obtained one blank-corrected sample measurement every 14 minutes and one standard solution measurement every 28 minutes during deployment. For NO_2_^-^ measurements, the off-chip Cd column was replaced with a short tube. The sensor operated the same as ΣNO_x_ sensor, with the only exception that the standard solutions of 0.5 and 2 μM NO_2_^-^ were used.

## Results and Discussion

### First tests of the LOC analyzer at elevated depth

**Fig 1:**
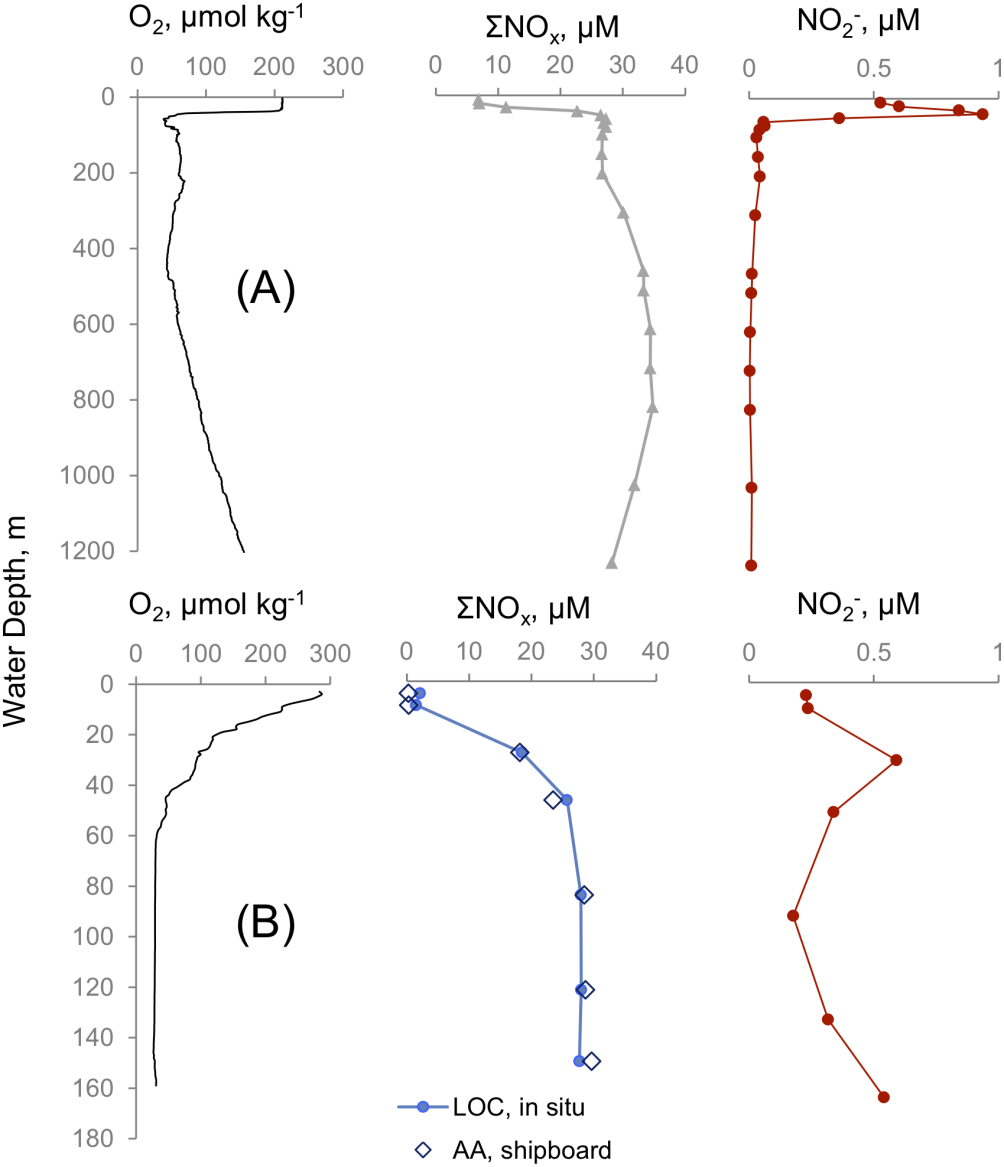
Water column chemistry in two stations in the Mauritanian OMZ (A) Water column profile of oxygen, NO_3_^-^+ NO_2_^-^ (ΣNO_x_) and NO_2_^-^ during a CTD cast (#15) at 1200 m depth. Both casts were conducted on June 12, 2014 at 16°48.32' W and 18°09.00' N. (B) Profiles from CTD cast #16 at a depth of 160 m where a LOC sensor was also attached to the CTD frame. “AA” stands for autoanalyer. These profiles were obtained on June 12, 2014 at 18°14.0' W and 16°31.0' N.

Two mid-depth O_2_ minima, sharp nitracline (increasing nitrate concentration over a relatively small depth range in the water column) and a subsurface NO_2_^-^ peak were persistent features in different CTD casts at the Mauritanian Shelf (one example shown in Fig 1A). Before the Lander deployments, we assessed the quality of the LOC sensor to reproduce vertical nutrient gradients by comparing it to concentrations obtained from water samples. It was therefore attached to the CTD frame during a profile to a depth of 160 m (CTD #16, S1 Fig). The sensor started to perform two sets of calibrations while the CTD was being lowered to 160 m. During the upcast, the rosette was stopped for 14 minutes at 7 different depths to allow for the in situ analysis of the blank (or a standard) and a sample. In the final minute of this period, a Niskin bottle sample was taken. Overall, the ΣNO_x_ distribution obtained from the LOC agreed well with the distribution of ΣNO_x_ obtained from shipboard autoanalyzer (AA) water samples (Fig 1B). All profiles showed that ΣNO_x_ was depleted at the surface with a nitracline located at about 20–40 m depth, below which ΣNO_x_ increased to about 30 μM. The location of the nitracline coincided with a NO_2_^-^ concentration peak of 0.6 μM at 30 m, which was also the upper boundary of the O_2_ minimum (29 μM after 60 m) extending down to 160 m.

### Analytical Uncertainty Assessment

**Fig 2:**
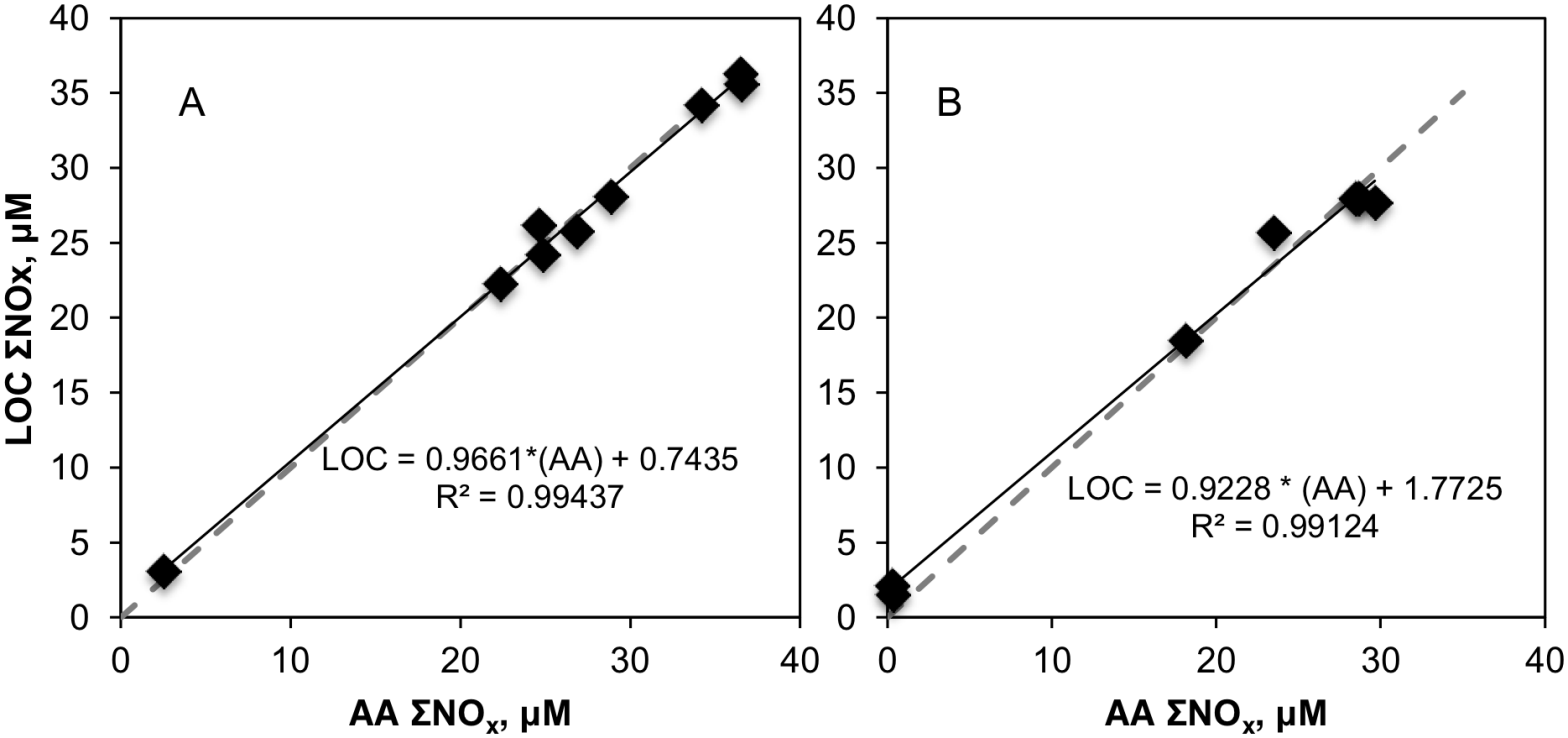
Comparison of autoanalyzer (AA) and LOC measurements (A) Autoanalyzer and LOC ΣNO_x_ measurements on samples obtained from hydrocast CTD#2, from 1000 m. Here, LOC was operated on a shipboard laboratory bench. (B) Autoanalyzer and in situ LOC ΣNO_x_ measurements on samples obtained from hydrocast CTD#16, from 170 m.

In addition to the submerged LOC tests during hydrocasts, we have performed additional comparisons (LOC measurement also shipboard) on bottle samples from another hydrocast at 1000 m (CTD#2). The combined scatter plots showing autoanalyzer (AA) and LOC measurements during CTD#2 and 16 (Fig 2) indicate that the results from the two approaches are highly correlated (R^2^ > 0.99). The differences at high (>5 μM) concentrations were comparable to the estimated uncertainties of the two approaches, which were calculated from two times the standard deviations of successive calibrations (on board for autoanalyzer and in situ for LOC). This analysis yielded a value of ±0.1 μM for the autoanalyzer and ±0.4–1 μM for the LOC analyzer (summarized in Table 2). Considering these inherent analytical uncertainties with the differences in sample handling by different operators, we can assert that the LOC nutrient sensor was capable of delivering in situ measurements at elevated depth with an analytical performance only slightly less precise than the established techniques.

**Table 2:** Summary of the analytical performance derived from in situ measurements of standard solutions (for LOC) and on board measurements (Autoanalyzer).

Series	Calibration solution, μM	Number of measurements	% Standard deviation from mean *	Estimated uncertainty, μM **
170 m ΣNO_x_	33.9	46	0.6	0.40
100 m ΣNO_x_	11.3	85	4.3	0.98
50m ΣNO_x_	11.3	63	3.4	0.76
50m NO_2_^-^	2.0	63	1.6	0.08
Autoanalyzer	5.8	10	0.8	0.10

* Average value of the moving (5) standard deviations

**Fig 3:**
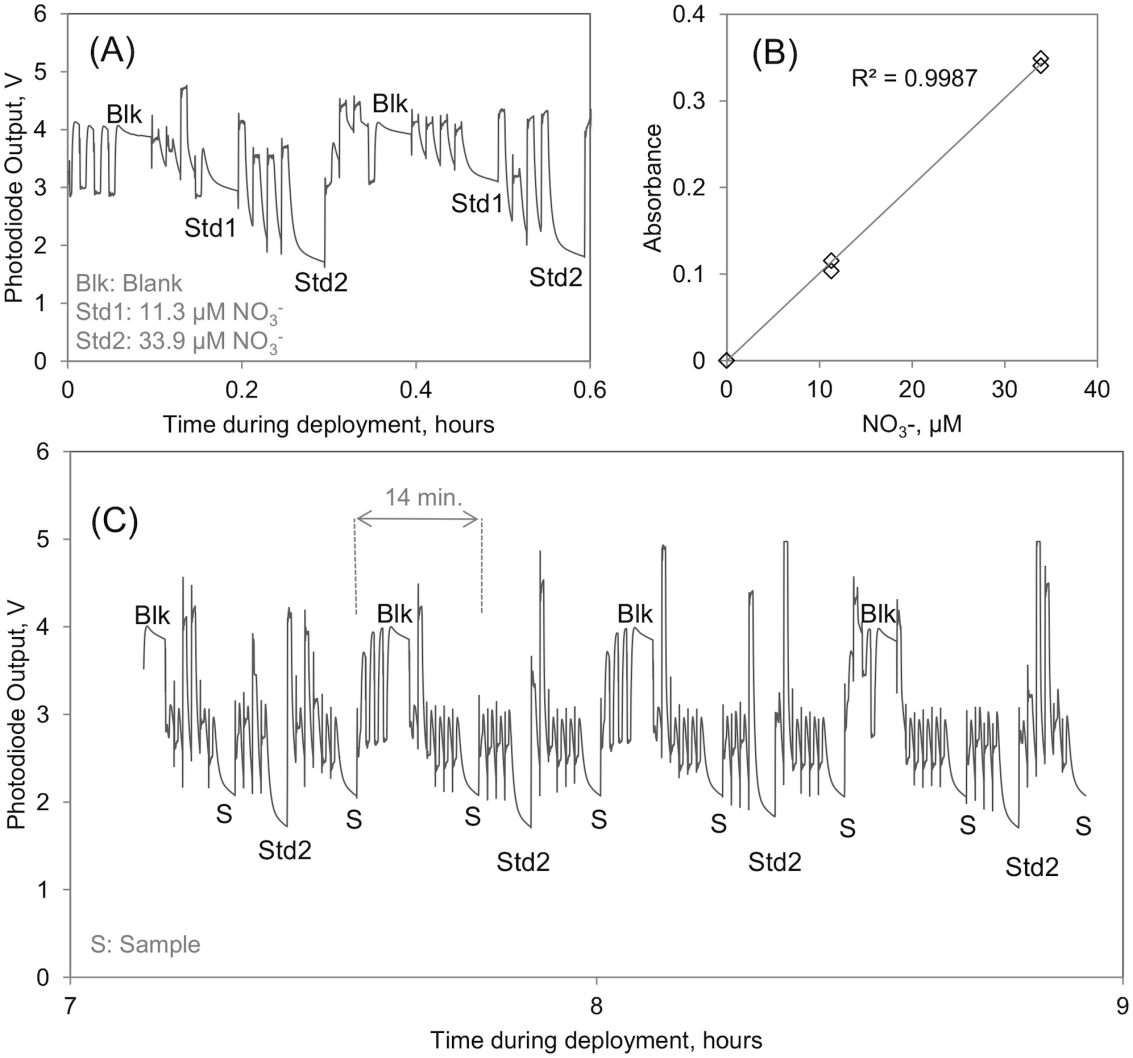
An example of the raw output of the lab-on-chip nitrate sensor. (A) Photodiode output showing the two sets of calibration performed in situ in the beginning of the series at 170 m depth. After the measurement of a blank (Blk), two standards solutions of NO_3_^-^ (Std1 and Std2) were measured and the calibration plot in (B) was obtained. Panel (C) shows an excerpt of the photodiode output during the same time series at 170 m depth, 7–9 hours after the first measurement. Blank solutions and the 33.9 μM standard solution were regularly measured in between sample (S) measurements, The fluctuating voltages were recorded during the flushing of the microfluidic chip and do not affect the measurements themselves.

**Fig 4:**
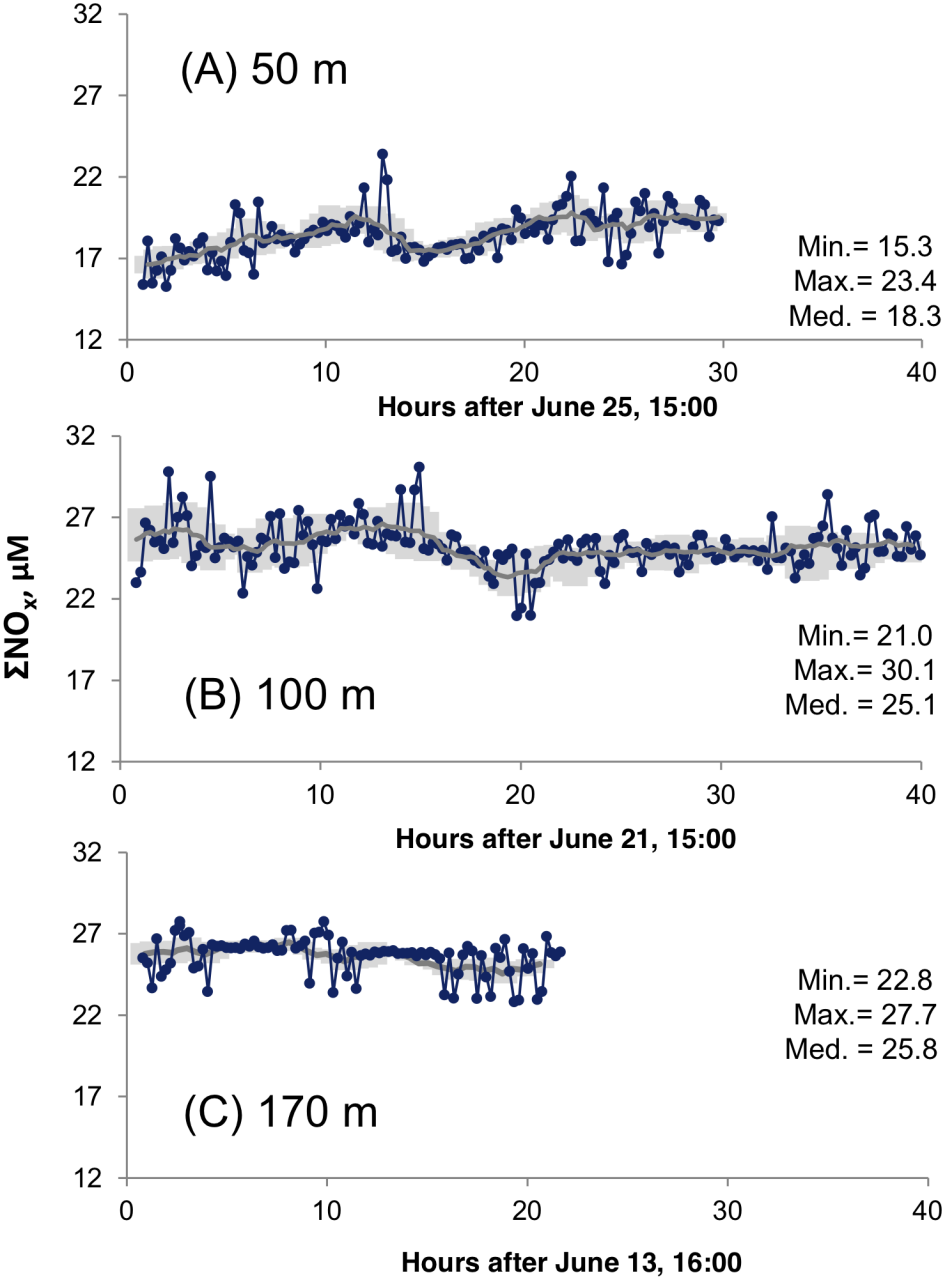
Time series measurements of NO3-+ NO2- (ΣNO_x_) in bottom waters. The time axis indicates time starting from the first sensor measurement in each respective deployment (see Table 1 for dates). The gray line in each series denotes a 12-point moving average. Also plotted as gray shading is ‘moving uncertainity’ corresponding to concentrations calculated from two times the moving (n = 5) standard deviations of the successive in situ measurements of a standard solution (Table 2). Any point outside the gray area very likely reflects natural variability.

** Calculated as the average of concentrations corresponding to 2 times the moving (5) standard deviations. These uncertainties are also shown on Figs 3 and 4.

In addition to the water sample comparison to validate sensor performance, it was also verified that the sensor performed reproducible in situ calibrations throughout all deployments, essential to obtain reliable time series data. An excerpt of raw photodiode readings featuring in situ blank, standard and sample measurements during the deployment at 170 m is shown in Fig 3. During all deployments no significant drift in the in situ calibrations was observed, however a random variability was present between successive calibrations from which sensor uncertainties were estimated (Table 2). The one blank-corrected sample per 14-minute measurement frequency used in this work should be considered as a minimum because we have aimed to fully utilize the in situ calibration feature and we had to use relatively high number of 6 flushing cycles due to high NO_3_^-^ concentrations. Although we have not investigated a full optimization, we think that the frequency can increase to 1 measurement per 10 minutes with less frequent blank/standard analyses. Using a smaller number of flushing cycles can further increase the sample analysis frequency for low concentration samples (<5 μM). Use of newly emerging microfluidic architectures such as multiplexed stop flow [42] can enable multiple simultaneous measurements on the same chip, increasing the frequency to one sample per minute or more.

### Nitrate and nitrite dynamics in the bottom boundary layer

The CTD casts only provided a snapshot of a dynamic system while the time series for ΣNO_x_ from the bottom waters at 50, 100 and 170 m (Fig 4) revealed high variability. The 50 m site had the lowest ΣNO_x_ concentrations (15.2 to 23.4 μM) while the 100 m site had a larger ΣNO_x_ range (21 to 30.1 μM) recorded in 40 hours. Over a 20-hour period, the 170 m site had ΣNO_x_ levels ranging from 22.8 to 27.7 μM, with 5–8 hours of very stable levels interrupted by more variable values. The median values of the time series measurements increased with depth, consistent with the trend suggested by the water column profiles. While the long-term (hours) variability was remarkable (discussed in detail in the next section), short-term variability on time scales less than hour was also high in certain periods. To compare the inherent sensor precision with natural variability, we have also plotted a ‘moving uncertainty’, calculated from two times the moving (n = 5) standard deviations of the successive in situ measurements of a standard solution (Fig 4 and Table 2). This comparison showed that most of the short-term variation less than a micromolar could be attributed to sensor response. However at certain periods there were marked deviations from the expected uncertainty, such as those in the 170 m time series (Fig 4C). The most likely physical explanation for the short-term ΣNO_x_ variability is the propagation of bores and non-linear internal wave trains, observed at the upper continental slope and the shelf off Mauritania at 18°N [19]. They are generated by interaction of barotropic and low-mode baroclinic tides with topography [43] and propagate onshore. Individual non-linear internal waves within the bores exhibited periods of 10 to 15 minutes and associated vertical velocities exceeded 0.15 m s^-1^ while on the continental slope they were particularly pronounced between 75 and 200m depth [19]. During their passage, they vertically displace water over a depth range of more than 70m. Thus we hypothesize that the short-term variability was due to the downward displacement of water having lower ΣNO_x_ concentrations from above. Non-linear internal waves have been observed on continental slopes in many continental slopes in the world ocean [44]. We suggest that they cause elevated high-frequency variability of near-bottom ΣNO_x_ concentrations and other solutes having vertical concentration gradients in the lower 50m to 100m of the water column, which may affect biogeochemical processes in the sediments and within the bottom boundary layer.

**Fig 5:**
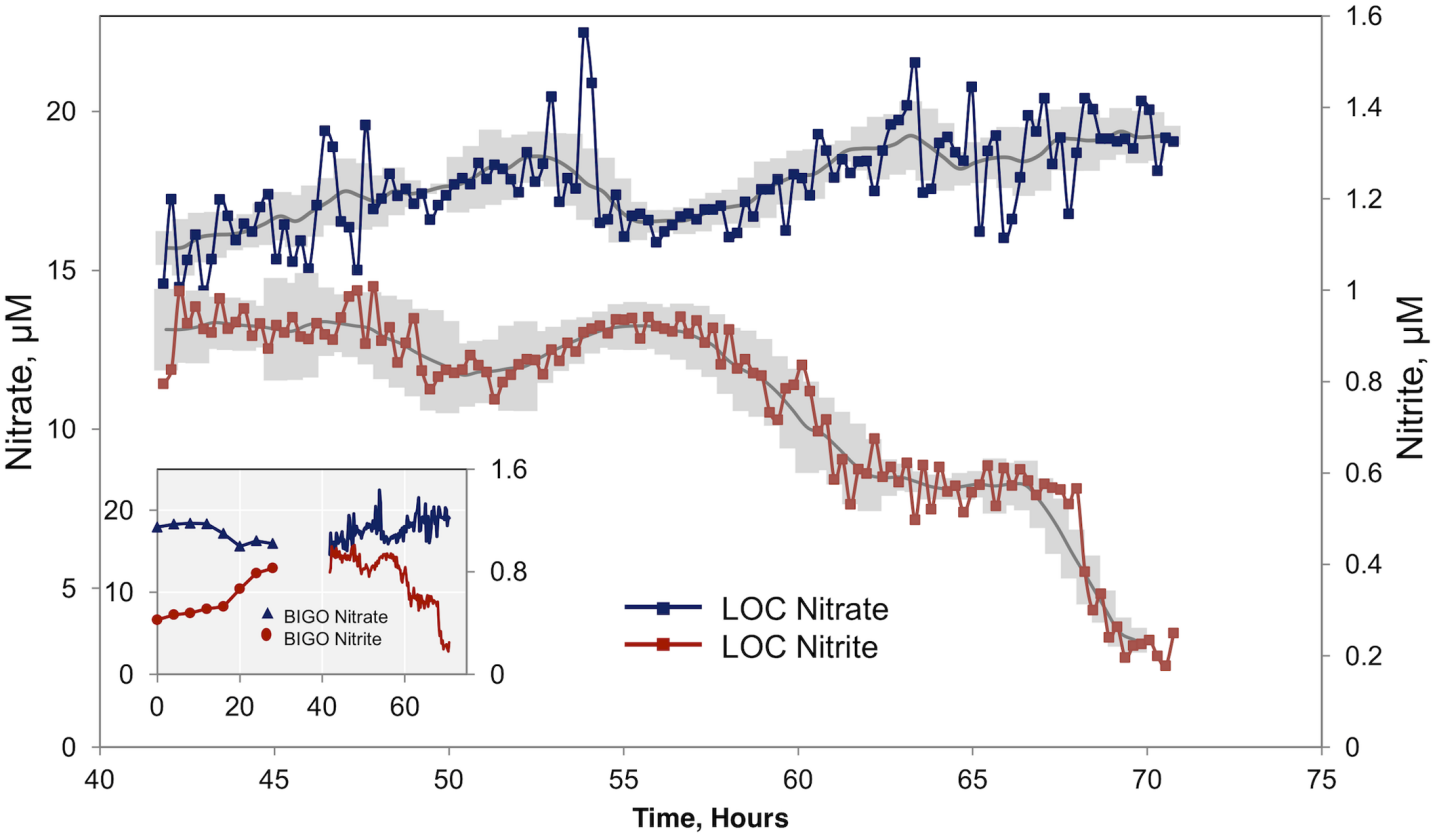
Simultaneous measurements of NO_3_^-^ and NO_2_^-^ at 50 m depth. Gray lines indicate 12-point moving averages. Also plotted as gray shading are ‘moving uncertainities’ calculated from two times the moving (n = 5) standard deviations of the successive in situ measurements of a standard calibration solution. The data from bottom water samples from a previous lander deployment (BIGOII-4) at this depth are also given in the inset. The time zero is set at the beginning of the BIGOII-4 deployment. When the time series from the lab-on-chip sensor are appended to the BIGOII-4 data (inset), a 3-day cycle emerges where the original NO_2_^-^ poor, NO_3_^-^ rich bottom water is replaced by water of higher NO_2_^-^ and lower NO_3_^-^. The water chemistry returns to original situation at the end of the 72-hour series.

At 50m, a location near the subsurface NO_2_^-^ peak (Fig 1), we deployed a second LOC system that measured NO_2_^-^ only in addition to the LOC measuring ΣNO_x_. The real-time clocks of the two sensors were synchronized, which enabled measurement of NO_2_^-^ simultaneously with NO_3_^-^ via subtracting NO_2_^-^ from ΣNO_x_. In the series, shown in Fig 5, NO_2_^-^ decreased from 1 μM to about 0.2 μM within 30 hours. Nitrate increased through the series with decreasing NO_2_^-^, suggesting a different water mass moved in, possibly originating from deeper waters where NO_3_^-^ is high and NO_2_^-^ is low. Remarkably, a similar magnitude of variability in the NO_3_^-^ NO_2_^-^ relationship was documented by measurements of bottom water samples sequentially taken via another benthic lander deployment (BIGOII-4, see Table 1) at the same position measuring benthic fluxes. This was deployed for two days prior to the Lander deployment and stopped sampling about 13 hours before the LOC sensors started measuring at the same depth. When LOC measurements are appended to the results of the discrete bottom water measurements (Fig 5, inset), a more complete picture emerges where the starting NO_2_^-^ poor, NO_3_^-^ rich waters were replaced by waters higher in NO_3_^-^ and lower in NO_2_^-^ through the middle of series. At the end of this period, the bottom water chemistry returns to the starting composition, suggesting a ~3 day cycle.

### Drivers of the long-term (hours) benthic biogeochemical variability

**Fig 6:**
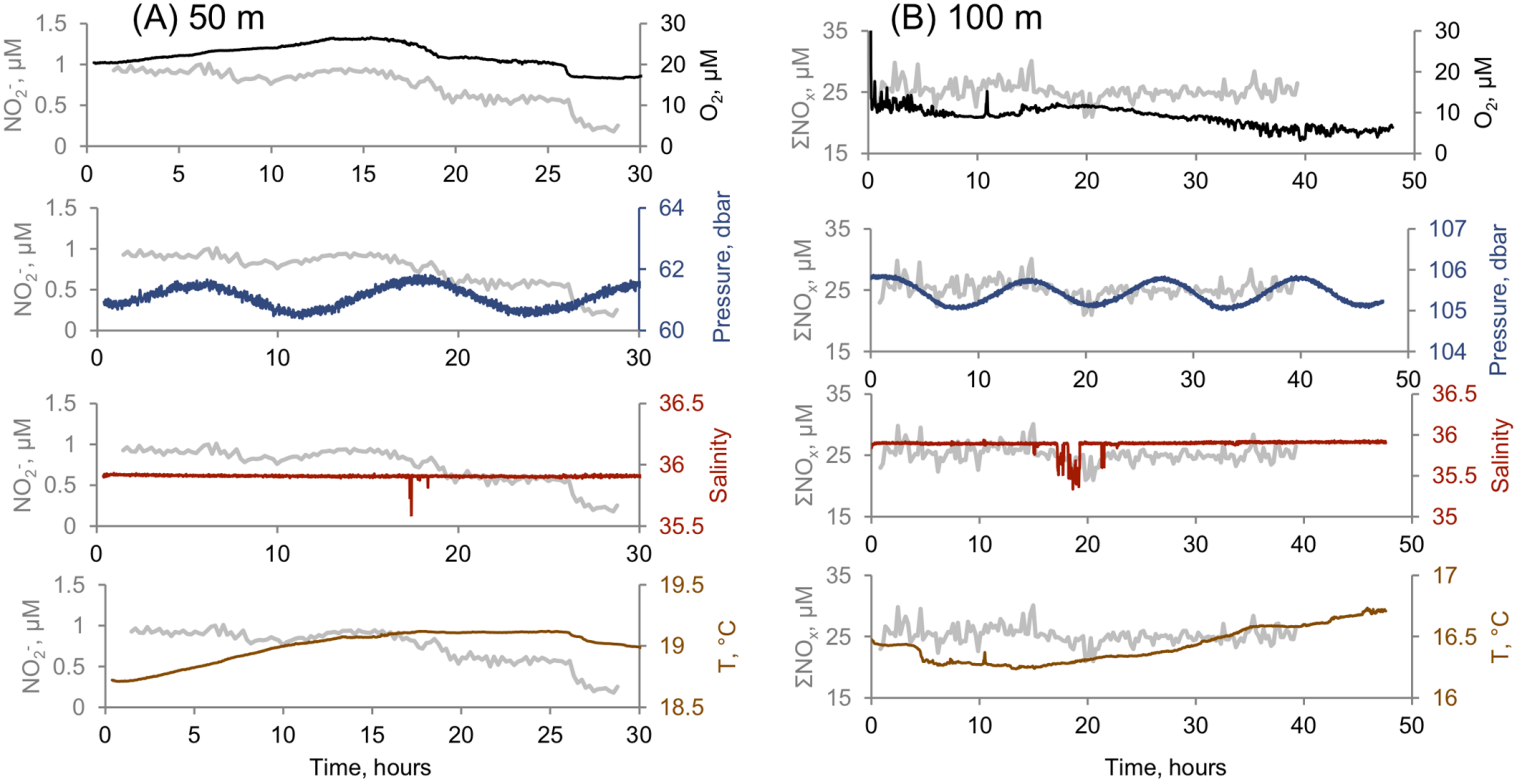
Time-Series of other parameters obtained during the lander deployments. (A) Time series of O_2_, pressure, salinity and temperature (black) plotted with NO_2_^-^ series (gray) in the 50 m site. (B) Time series of O_2_, pressure, salinity and temperature (black) plotted with ΣNO_x_ series (gray) in the 100 m site.

In order to account for the observed variability of nutrients over several hours, we also recorded physical parameters and oxygen at the 50 and 100m sites (Fig 6). In both sites, the tides did not explain any of the nutrient variability. Cross correlation matrices of the series (S2 Fig) revealed that the 50 m site had more correlated pairs than the 100 m site. Considering this difference, and the fact that our dataset is more complete for the 50 m site, we focus our discussion on the 50 m site. At the 50 m series, the NO_2_^-^, which correlated inversely with NO_3_^-^ (Spearman Rank Correlation, r = -0.59, p<0.01), also had a significant inverse correlation with temperature (T) (r = -0.52, p<0.01) and positive correlation with O_2_ (r = 0.58, p<0.01). Nitrate had weaker, but still significant correlations (in opposite sign to that of NO_2_^-^) with these parameters. Taken together, these findings suggest that during the course of the 50m time series NO_3_^-^ rich NO_2_^-^ -poor cooler waters replaced the original warmer, O_2_-rich, NO_2_^-^ rich waters. Therefore the overall trend was towards increasing temperatures, decreasing NO_2_^-^ and O_2_ while increasing NO_3_^-^ (Fig 4 and Fig 6a).

**Fig 7:**
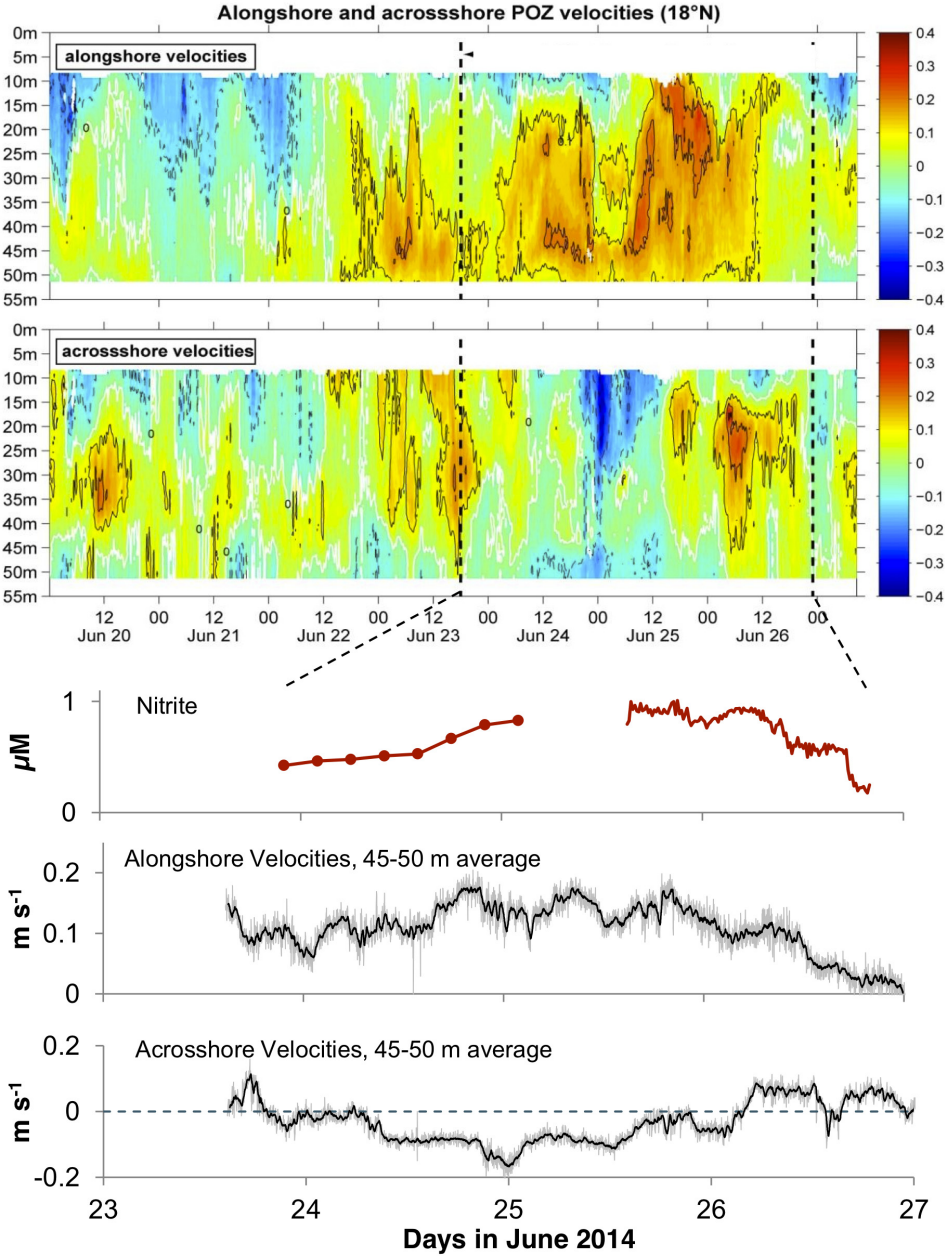
Alongshore and across shore velocities (m s^-1^) at the 50 m site. The velocities were measured with an upward-looking ADCP on a lander at 50m on the 18° N transect. Positive alongshore velocities values stand for northward flow while positive across shore velocities represent flow towards the shore. The NO_2_^-^ (μM) series shown in Fig 4 are also added to indicate that the high- NO_2_^-^ periods were associated with strong positive alongshore velocities and negative across shore velocities.

What drives these non-tidal variations in the chemical and physical parameters? To attempt an explanation we examined the velocity profiles from an acoustic Doppler current profiler (ADCP) that was deployed on the sea floor at the depth of 50 m, close to the Lander deployment site (Table 1). Over a 15-day period, the alongshore and across-shore currents fluctuated significantly reaching velocities of 0.4 m s^-1^ (Fig 7). Close to the sea floor at 45–50 m depths, a notable northward flow existed with intermittent onshore and offshore flow. Focusing on the period of chemical time series at 50 m (June 23–27 2014, Fig 7); we found that the high- NO_2_^-^ low- NO_3_^-^ period coincided with the maximum northward alongshore flow in much of the water column and elevated offshore flow in the near bottom layer. When the alongshore flow decreased on June 26, an onshore flow in the near bottom layer became pronounced attaining velocities up to 0.1 m s^-1^. The change in the current direction is coincident with the steep decrease in NO_2_^-^, and indicates deep-water movement towards the shore.

The correlation between near-bottom onshore flow variability and alongshore flow variability in the water column can be interpreted in terms of bottom-boundary layer Ekman dynamics. Due to bottom friction and the Coriolis force, a northward alongshore flow above the bottom boundary layer will generate an offshore flow component in the bottom boundary layer, which is in agreement with velocity time series (Fig 7). Thus, during the period of elevated northward flow on the shelf, the intrusion of deeper waters was blocked and water masses from the shallower shelf regions that were low in NO_3_^-^ and high in NO_2_^-^ were advected offshore. When the northward current ceased, deeper waters started to move on shore, which resulted in higher NO_3_^-^, lower NO_2_^-^ and lower O_2_ concentrations (Fig 6). While the northward flow on the shelf can generally be attributed to the Mauritanian current during this period [32], the elevated northward velocities between June 22 and June 26 represent an intermittent feature that is probably due to local wind forcing. One biogeochemical implication is that these fluctuating currents may lead to periodic pumping of deep waters at the 50 m site. At this shallow site this might be an important mechanism for nutrient transport to support primary production as well as for the supply of electron acceptors to drive denitrification and the subsequent nitrogen redox process in the upper sediments. While the hydrodynamically-driven variability in NO_3_^-^ concentrations is pronounced in the seafloor of the Mauritanian Upwelling, the variability is even more dramatic for NO_2_^-^ when relative changes are considered (Fig 5). At the 50 m site, the bottom water NO_2_^-^ concentration decreased fivefold, from 1 μM to 0.2 μM in just 30 hours. Porewater NO_2_^-^ concentrations in the surface sediments at this site were in the order of several hundred nanomolars (ref. [17] and unpubl. data from M107). Hence, the dramatic change in the bottom water NO_2_^-^ concentration can influence the direction and magnitude of the benthic NO_2_^-^ fluxes. Future longer-term deployments with a network of nutrient sensors can answer questions such as whether or not this dramatic variability in NO_2_^-^ exists at other sites and how this variability should be accounted for in determining the benthic biogeochemical feedback in this productive coastal ocean.

## Conclusions and Future Perspectives

We have successfully deployed a new prototype microfluidic lab-on-chip (LOC) NO_3_^-^/ NO_2_^-^ sensor in the bottom waters of a productive coastal ocean. To our knowledge this is the first report of time series from a LOC NO_3_^-^/ NO_2_^-^ sensor in a deep underwater setting. We found that the analytical performance of the sensor at elevated depth was comparable to a conventional autoanalyzer. Coupled to time series measurements of oxygen and currents, the LOC measurements recorded large variations in nutrients, probably linked to cross-shelf water transport occurring in relation to the dynamics of the alongshore flow on the Mauritanian shelf. Relative variability of NO_2_^-^ in the bottom waters was larger and this could affect the magnitude and direction of benthic biogeochemical fluxes in this low-oxygen coastal ocean.

Although we have not tested the LOC sensor in situ at depths beyond 170 m, our results show that the microfluidic sensor approach is not compromised by elevated pressures and therefore has a high potential for deep-water applications. New developments in sensor technology (such as miniaturization and new microfluidic architectures) are expected to decrease the size of these devices, allow the introduction of new chemical parameters, increase data acquisition frequency and enhance the applicability of the sensor towards Lagrangian (moving) platforms. These developments will not only be beneficial to Earth observation but also to space and planetary exploration—indeed this motivation was the starting point of our sensor testing work under the framework of Helmholtz Alliance ROBEX (Robotic Exploration of Extreme Environments) [45]. Particularly, having robust sensors at high technological maturity for underwater applications could contribute to future missions to active icy moons such as the Jovian moon Europa and Enceladus in the Saturnian system [46]. As the potential habitability of these moons is receiving increasing attention; the detection of biosignatures, dissolved nutrients or energy-yielding chemicals in the subsurface oceans [47] of these icy satellites would considerably benefit from in-situ analysis. In order to support this vision, and more specifically to increase the technological readiness of the new sensors, we need to continue to bring new generation chemical sensors to extreme environments such as deep sea, assess their capabilities and limitations and eventually implement sensor networks that will pave the way for more extensive testing and the much-needed highly resolved in situ biogeochemical datasets.

## Supporting Information

S1 Fig The LOC sensor as attached to the CTD rosette and the benthic lander. (TIFF)

S2 Fig Cross correlation matrices for the multi-parameter datasets obtained from 50 and 100m. Units are μM for NO_2_^-^, NO_3_^-^ and O_2_; dbars for pressure and °C for temperature. Stars indicate significant correlation (p<0.01) between the pair.
(TIFF)

S1 File All data from the LOC sensors and autoanalyzer measurements on bottle samples. (ZIP)

S2 File All data from the POZ landers on the current measurements in the bottom waters. (ZIP)
