# Supplementary figures and images for: Nitrate and Nitrite Variability at the Seafloor of an Oxygen Minimum Zone Revealed by a Novel Microfluidic In-Situ Chemical Sensor
Source: PLoS One. 2015 Jul 10;10(7):e0132785. doi: 10.1371/journal.pone.0132785 (PMC4498834; doi:10.1371/journal.pone.0132785)

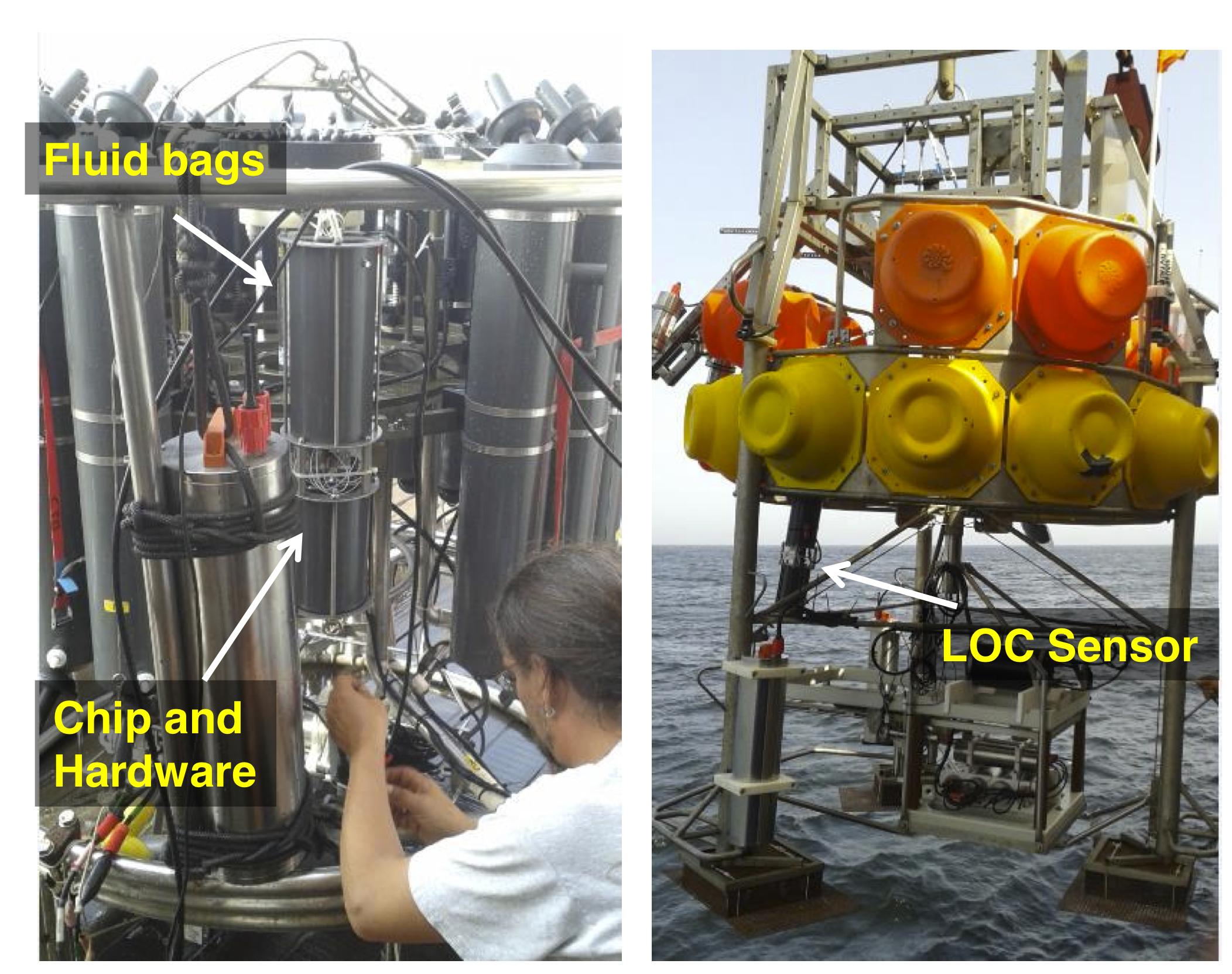

Supplement: S1 Fig — (TIFF) [file pone.0132785.s001.tiff]

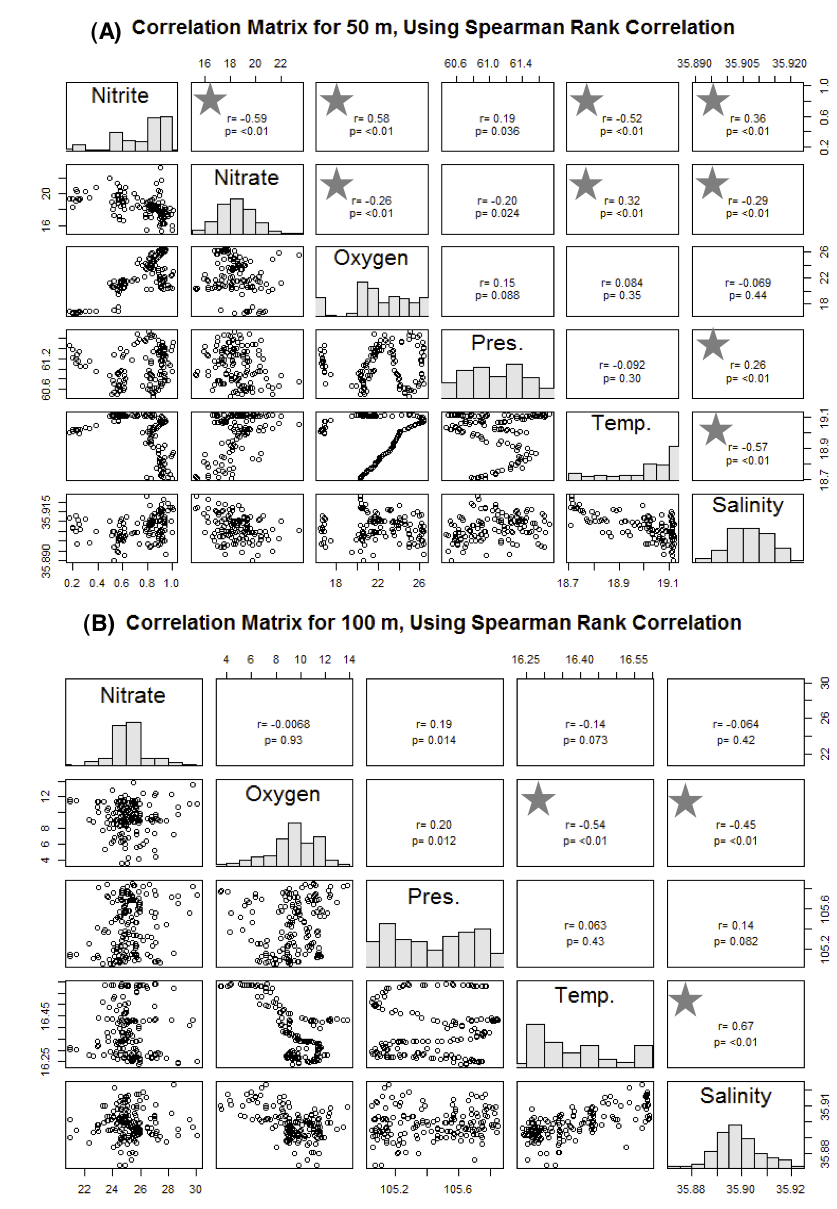

Supplement: S2 Fig — Units are μM for NO2 -, NO3 - and O2; dbars for pressure and °C for temperature. Stars indicate significant correlation (p<0.01) between the pair. (TIFF) [file pone.0132785.s002.tiff]
